# *Helicobacter pylori* Infection and Metachronous Gastric Cancer in Elderly Patients With Gastric Cancer Aged ≥ 75 Years Who Underwent Endoscopic Submucosal Dissection

**DOI:** 10.1111/hel.70068

**Published:** 2025-09-01

**Authors:** Young‐Il Kim, Jong Yeul Lee, Chan Gyoo Kim, Il Ju Choi

**Affiliations:** ^1^ Center for Gastric Cancer, National Cancer Center Goyang Korea

**Keywords:** elderly, endoscopic submucosal dissection, *Helicobacter pylori*, metachronous gastric cancer

## Abstract

**Background and Aims:**

*Helicobacter pylori* (*Hp*) infection is associated with metachronous gastric cancer (GC) after endoscopic submucosal dissection resection (ESD) in patients with early GC (EGC), but this association has not been well investigated in elderly patients. This study investigated whether *Hp* infection status was associated with metachronous GC after ESD in patients aged ≥ 75 years.

**Methods:**

This retrospective study involved 298 EGC patients aged ≥ 75 years who underwent ESD. The *Hp*‐negative group (*n* = 233) included patients with negative or eradicated *Hp* infection, whereas the *Hp*‐positive group (*n* = 65) included patients with persistently positive infection or failed eradication. The primary outcome was metachronous GC occurring at ≥ 1 year after ESD.

**Results:**

The median patient age was 78 years (interquartile range [IQR]: 76–80 years). During a median follow‐up of 4.4 years (IQR: 2.9–5.9 years), metachronous GC occurred in 16 (6.9% [16/233], 16.3 cases/1000 person‐year) and 10 (15.4% [10/65], 37.5 cases/1000 person‐year) patients in the *Hp*‐negative and *Hp*‐positive groups, respectively. The incidence of metachronous cancer was higher in the *Hp*‐positive group than in the *Hp*‐negative group (*p* = 0.035, log‐rank test). In a multivariate analysis, persistent *Hp* infection was an independent risk factor for metachronous GC (age‐ and sex‐adjusted hazard ratio, 2.33; 95% CI: 1.05–5.17).

**Conclusions:**

Persistent *H. pylori* infection status was associated with a higher risk of metachronous GC, and *H. pylori* treatment needs to be provided in elderly patients aged ≥ 75 years and older with EGC undergoing ESD.

## 1 Introduction

Globally, life expectancy at birth was reported to be 73.3 years in 2024 and has increased by 8.4 years since 1995, owing to improvements in medical and nutritional healthcare [1]. With an increase in life expectancy, the population aged ≥ 65 years accounted for 9% in 2019 and is expected to reach 16% by 2050 worldwide [2]. The gastric cancer incidence increases with age (proportions of gastric cancer in 2022 worldwide, 63% in patients aged ≥ 65 years and 31% in patients aged ≥ 75 years) [3]. In early gastric cancer (EGC) patients, endoscopic submucosal dissection (ESD) is a minimally invasive and standard treatment for tumors that meet indications [4, 5]. Although about two‐thirds of elderly patients have multiple comorbidities associated with poor treatment outcomes [6], ESD is a safe and feasible treatment option, even in late elderly EGC patients aged ≥ 75 years [7, 8]. Thus, ESD is the preferred treatment over gastrectomy in elderly EGC patients, considering their physical condition and life expectancy.

Previous studies reported comparable overall survival (OS) in elderly EGC patients who underwent ESD and those who underwent gastrectomy [9, 10]. Patients who underwent ESD developed less severe treatment‐related adverse events (AEs) and had a lower proportion of treatment‐related AEs requiring surgery or intensive care unit admission than those who underwent surgery alone [9, 10]. Despite its advantages as a non‐invasive treatment with fewer AEs and comparable long‐term outcomes, metachronous gastric cancer (MGC) occurred with about 3% per year after ESD because of stomach preservation [9, 11]. Thus, *Helicobacter pylori* eradication is recommended to prevent MGC. A meta‐analysis including three randomized controlled trials (RCTs) reported that *H. pylori* therapy reduced the MGC risk by 51% after ESD in patients with EGC [12]. However, previous RCTs evaluated the MGC prevention effects of *H. pylori* therapy mainly involving younger EGC patients aged < 75 years [13, 14]. The preventive effects and association between *H. pylori* treatment and MGC have not been well evaluated in patients with EGC aged ≥ 75 years.

We investigated the association between *H. pylori* infection status and MGC in late elderly EGC patients (≥ 75 years) who underwent ESD.

## 2 Methods

### 2.1 Patients

This study was a retrospective cohort study. Patients aged ≥ 75 years who had undergone ESD for EGC from April 2002 to December 2020 were included. The following patients were excluded: patients with no information on the follow‐up *H. pylori* status, those who had a short‐term follow‐up (< 1 year), those who had a history of gastrectomy, and those with noncurative ESD on the final pathology according to the gastric cancer treatment guidelines [4, 5]. We retrospectively reviewed and collected patient data on demographics, clinical, and tumor characteristics. The Charlson comorbidity index (CCI) score was calculated to evaluate patient condition according to comorbid illnesses [15]. The study was approved by the institutional ethics committee (approval number, NCC2024‐0061), and informed consent from patients was waived due to the minimal risk of this study.

### 2.2 ESD Procedure and Endoscopic Evaluations at Baseline and Follow‐Up

At baseline, the endoscopic evaluation of EGC, the assessment of *H. pylori* infection was performed by histological evaluations of biopsy specimens, involving the rapid urease test (RUT) at the corpus greater curvature and Wright–Giemsa staining. In some patients, gastric atrophy (GA) and intestinal metaplasia (IM) were assessed using biopsy specimens obtained from the lesser curvature of the antrum and corpus. The operative link on gastritis assessment (OLGA) and gastric IM (OLGIM) stages was obtained using grades of GA and IM [16, 17].

Subsequently, the patients underwent ESD, as described previously [14, 18]. During post‐ESD periods, scheduled follow‐up endoscopic evaluations were carried out to detect cancer recurrence at 3, 6, and 12 months, and then every 6 months or annually. At the discretion of the endoscopists, the *H. pylori* infection status was assessed using RUT or histology during follow‐up endoscopic evaluations.

### 2.3 *H. pylori* Treatment

In Korea, patients with EGC should pay all the costs of *H. pylori* treatment after ESD before 2018. In addition to the payment issue, not all late elderly patients received *H. pylori* treatment because of several comorbidities, including cardiac diseases and renal or liver dysfunction, that could increase AEs due to *H. pylori* treatment regimens. The first‐line treatment regimen comprised a 7 to 14 day course of proton pump inhibitor‐clarithromycin‐amoxicillin triple therapy, and the second‐line treatment regimen was bismuth‐containing quadruple therapy, according to the Korean *H. pylori* management guidelines [19]. The success of *H. pylori* eradication was confirmed by a urea breath test 4 to 8 weeks after treatment.

### 2.4 Outcomes

The primary outcome was MGC occurrence, which was defined as gastric cancer detected at a previously uninvolved site 1 year or greater after ESD. Other outcomes included risk factors associated with the MGC and OS. The last follow‐up information on recurrence and death was obtained in April 2024. A subgroup analysis of MGC occurrence by the OLGA and OLGIM stages was performed in patients with data on GA and IM.

### 2.5 Statistical Analysis

Patients with negative *H. pylori* test and those with successfully eradicated *H. pylori* infection were included in the *Hp*‐negative group. Patients with a consistently positive *H. pylori* infection status and those with failed eradication were included in the *Hp*‐positive group. Student *t*‐test or Mann–Whitney *U* test was used for evaluating differences in continuous variables, and the chi‐square test or Fisher's exact test was used for categorical variables. Kaplan–Meier curves for metachronous recurrence of gastric cancer were plotted, and the log‐rank test was used to compare the curves. Risk factors associated with metachronous recurrence were evaluated using the Cox proportional hazard regression model. All statistical analyses were performed using STATA version 18.0 (StataCorp., TX, USA). A *p*‐value less than 0.05 was statistically significant.

## 3 Results

### 3.1 Patient Baseline Characteristics

**FIGURE 1:**
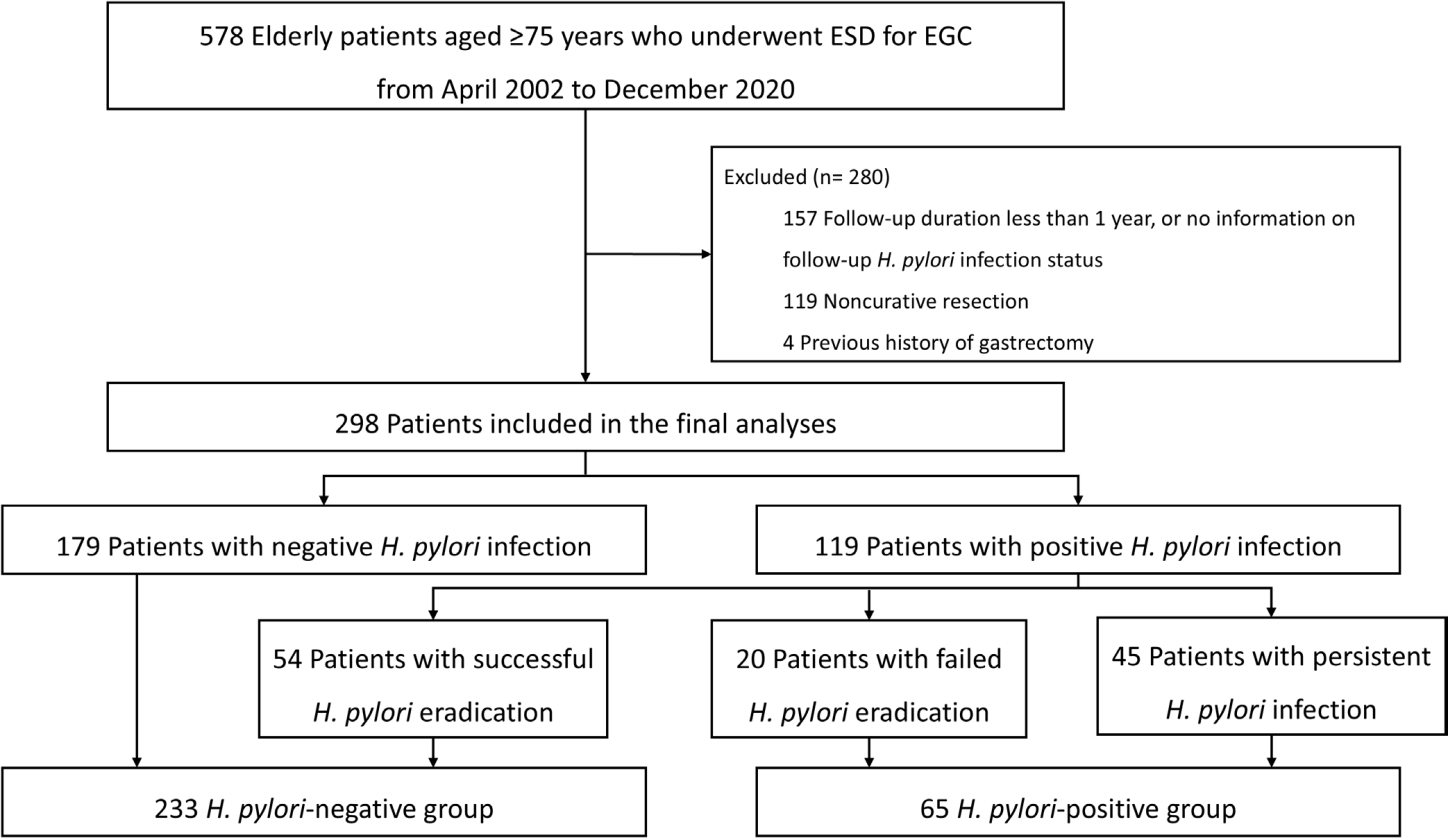
Study flows. EGC, early gastric cancer; ESD, endoscopic submucosal dissection.

Between April 2002 and December 2020, 578 older patients underwent ESD for EGC (Figure 1). Of these, 280 patients were excluded (157 patients with a follow‐up period of < 1 year or no information on follow‐up *H. pylori* infection status; 119, non‐curative resection on the final pathological evaluation; and history of gastrectomy). Finally, 298 patients were included (233 patients in the *Hp*‐negative group and 65 in the *Hp*‐positive group). The comparisons of baseline patient and tumor characteristics between the 280 excluded patients and 298 included patients are presented in Table S1.

The median patient age was 78 years (interquartile range [IQR]: 76–80 years; range, minimum 75 years to maximum 90 years). The baseline clinical and tumor characteristics are described in Table 1. Two or more comorbidities were present in 81 patients (27.2%), and the proportion of patients with CCI ≥ 2 was 30.5% (91 patients). Clinical characteristics, including age, sex, co‐morbid illnesses, CCI score, and antithrombotic drug use, did not differ according to the *H. pylori* status. The *Hp*‐positive group (9.2%) had a higher proportion of undifferentiated histological types than the *Hp*‐negative group (1.7%; *p* = 0.009). The tumor size, location, invasion depth, and initial multiple tumor proportions did not differ between the two groups.

**TABLE 1:** Baseline patient and tumor characteristics.

	Total	Group		*p*
		*H. pylori* ‐negative	*H. pylori* ‐positive	
	(*n* = 298)	(*n* = 233)	(*n* = 65)	
Age (year), median (IQR)	78 (76–80)	78 (76–80)	78 (77–80)	0.391
Sex, no (%)				
Female	97 (32.6)	75 (32.2)	22 (33.8)	0.801
Male	201 (67.4)	158 (67.8)	43 (66.2)	
Smoking, no (%)				
Never	148 (49.7)	115 (49.4)	33 (50.8)	0.840
Former or current	150 (50.3)	118 (50.6)	32 (49.2)	
Alcohol drinking, no (%)				
Never	182 (61.1)	144 (61.8)	38 (58.5)	0.625
Former or current	116 (38.9)	89 (38.2)	27 (41.5)	
Co‐morbid illness, no (%)				
Hypertension	158 (53.0)	128 (54.9)	30 (46.2)	0.210
Diabetes mellitus	65 (21.8)	52 (22.3)	13 (20.0)	0.689
Atrial fibrillation	12 (4.0)	10 (4.3)	2 (3.1)	0.660
Cardiovascular disease^a^	38 (12.8)	31 (13.3)	7 (10.8)	0.588
Chronic lung disease	5 (1.7)	4 (1.7)	1 (1.5)	> 0.999
Chronic liver disease	14 (4.7)	12 (5.2)	2 (3.1)	0.742
Chronic kidney disease	2 (0.7)	2 (0.9)	0 (0)	> 0.999
Other organ cancer	27 (9.1)	23 (9.9)	4 (6.2)	0.356
Two or more co‐morbid illnesses	81 (27.2)	68 (29.2)	13 (20.0)	0.141
CCI score, no (%)				
0	83 (27.9)	59 (25.3)	24 (36.9)	0.197
1	124 (41.6)	100 (42.9)	24 (36.9)	
≥ 2	91 (30.5)	74 (31.8)	17 (26.2)	
Family history of gastric cancer, no (%)	50 (16.8)	44 (18.9)	6 (9.2)	0.066
Use of antithrombotic drugs, no (%)				
No	215 (72.1)	164 (70.4)	51 (78.5)	0.446
Antiplatelet drugs	79 (26.5)	65 (27.9)	14 (21.5)	
Anticoagulant	4 (1.3)	4 (1.7)	0 (0)	
Tumor size (cm), mean ± SD	1.9 ± 1.2	1.9 ± 1.2	1.8 ± 0.9	0.546
Tumor location, no (%)				
Upper	34 (11.4)	29 (12.4)	5 (7.7)	0.370
Middle	81 (27.2)	66 (28.3)	15 (23.1)	
Lower	183 (61.4)	138 (59.2)	45 (69.2)	
Tumor depth, no (%)				
Mucosa	247 (82.9)	190 (81.5)	57 (87.7)	0.245
Submucosa	51 (17.1)	43 (18.5)	8 (12.3)	
Tumor histologic type, no (%)				
Differentiated type	288 (96.6)	229 (98.3)	59 (90.8)	0.009
Undifferentiated type	10 (3.4)	4 (1.7)	6 (9.2)	
Initial multiple tumors, no (%)	32 (10.7)	28 (12.0)	4 (6.2)	0.177

Abbreviations: CCI, Charlson comorbidity index; IQR, interquartile range; SD, standard deviation.

^a^ Cardiovascular diseases include coronary artery diseases (angina pectoris, myocardial infarction) and cerebrovascular diseases (cerebral hemorrhage and infarction).

### 3.2 Metachronous Gastric Cancer Recurrence

**FIGURE 2:**
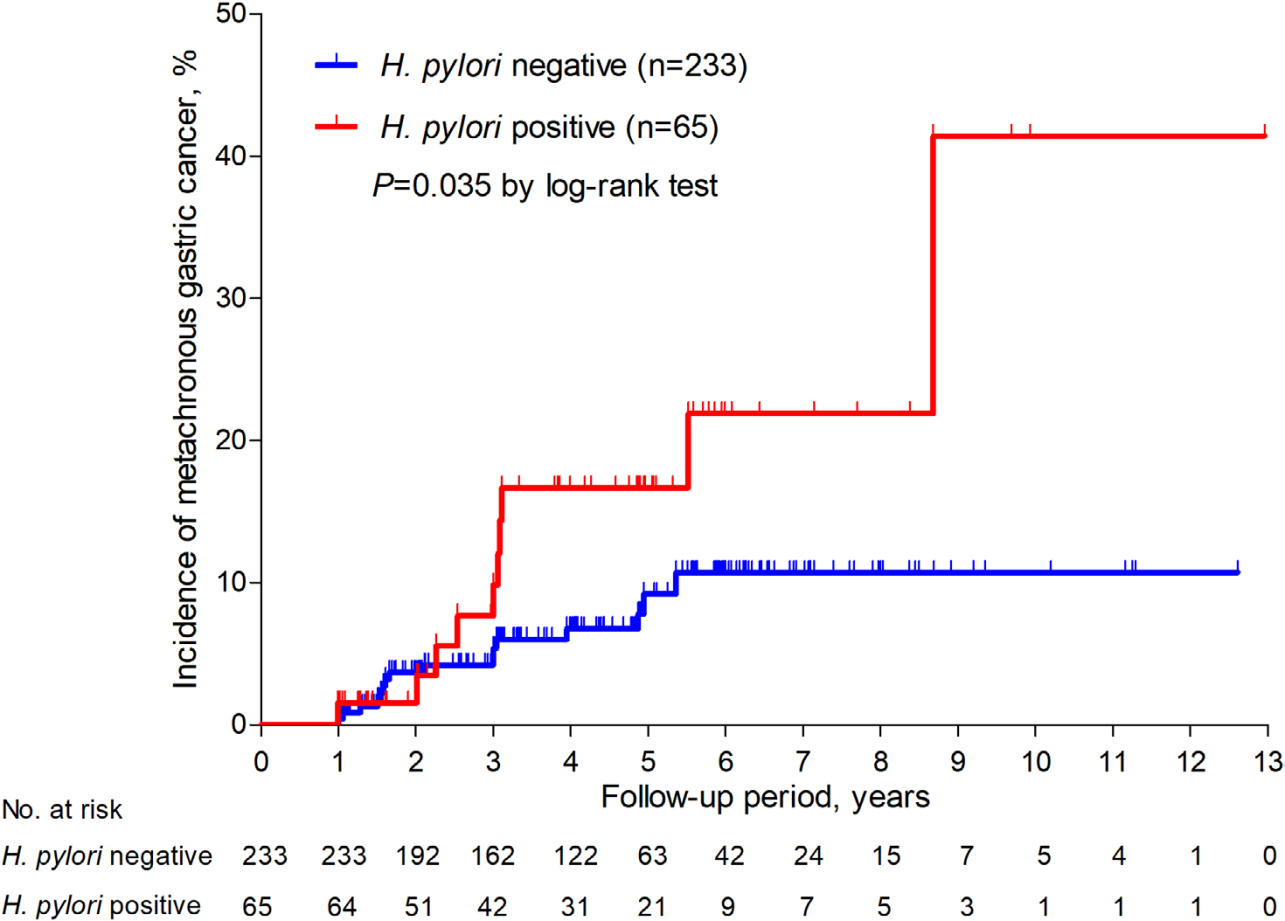
Incidence of metachronous gastric cancer according to the *H. pylori* infection status.

The duration of follow‐up periods did not differ between the *Hp*‐negative group (median 4.3 years; IQR: 3.0–5.9 years) and the *Hp*‐positive group (median 4.8 years; IQR: 2.2–6.0 years) (*p* = 0.692). The MGC was detected in 16 patients (6.9% [17/233 patients]) in the *Hp*‐negative group and in 10 patients (15.4% [10/65 patients]) in the *Hp*‐positive group. The *Hp*‐positive group (37.47 cases/1000 person‐year) showed a significantly higher incidence of MGC than the *Hp*‐negative group (16.29 cases/1000 person‐year; *p* = 0.035 by log‐rank test) (Figure 2). In a subgroup analysis, patients with *H. pylori* ‐persistent infection had a higher incidence of MGC than those with *H. pylori* ‐eradicated infection (7.4% [4/54 patients]; 16.91 cases/1000 person‐year), which approached statistical significance (*p* = 0.082 by log‐rank test) (Figure S1).

In the *Hp*‐negative group, no statistically significant difference was observed between the patients with negative *H. pylori* infection (6.7% [12/179 patients]; 16.09 cases/1000 person‐year) and those with eradicated status (7.4% [4/54 patients]; 16.91 cases/1000 person‐year) (*p* = 0.973 by log‐rank test) (Figure S2).

### 3.3 Metachronous Recurrence of Gastric Cancer: Patient Characteristics and Treatment

The patient characteristics and treatment for MGC are presented in Table S2. The median time to MGC occurrence after ESD was 2.8 years (IQR: 1.6 to 3.1 years). The histologic type of all MGCs was the differentiated type, and most tumors (96.2%, 25 of the 26 cases) were confined to the mucosa, except for one case with submucosal invasion. Of the 26 patients with recurrence, 23 (88.5%) underwent ESD. One patient underwent argon plasma coagulation because of an underlying disease (stage IV lung cancer). The remaining two patients underwent surgery because of peri‐gastric lymph node metastasis on abdominal computed tomography.

**FIGURE 3:**
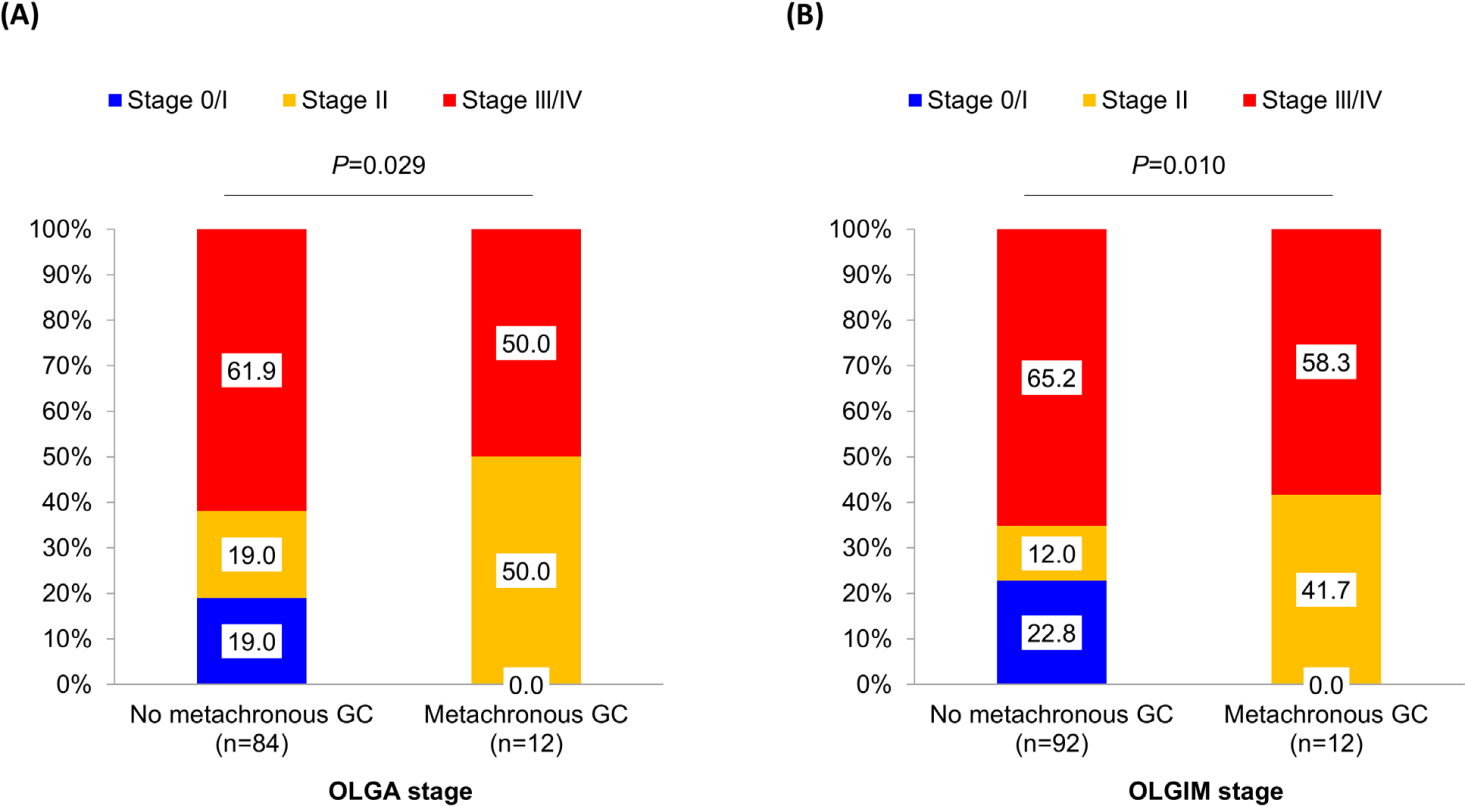
Distributions of the OLGA and OLGIM stages according to the metachronous gastric cancer occurrence.

Compared with patients without MGC, the baseline clinical and tumor characteristics of patients with MGC did not differ, except for a higher proportion of persistent *H. pylori* ‐positive infection status (20.2% vs. 38.5%; *p* = 0.031) (Table 2). Subgroup analyses were performed, including patients with information on the OLGA stage (*n* = 96) and OLGIM stage (*n* = 104) (Figure 3). A higher proportion of OLGA (*p* = 0.029) and OLGIM (*p* = 0.010) stages II–IV was observed in patients with MGC than in those without MGC. All MGCs occurred in patients with OLGA/OLGIM stages II to IV, whereas no MGCs were observed in patients with OLGA/OLGIM stages 0–I.

**TABLE 2:** Comparisons of patient and tumor characteristics according to the recurrence of metachronous gastric cancer.

	Metachronous gastric cancer		*p*
	No	Yes	
	(*n* = 272)	(*n* = 26)	
Age (year), median (IQR)	78 (76–80)	77 (76–80)	0.310
Sex, no (%)			
Female	89 (32.7)	8 (30.8)	0.839
Male	183 (67.3)	18 (69.2)	
*H. pylori* infection status, no (%)			
Negative or eradicated	217 (79.8)	16 (61.5)	0.031
Persistent	55 (20.2)	10 (38.5)	
Co‐morbid illness, no (%)			
Hypertension	141 (51.8)	17 (65.4)	0.186
Diabetes mellitus	59 (21.7)	6 (23.1)	0.870
Atrial fibrillation	10 (3.7)	2 (7.7)	0.320
Cardiovascular disease^a^	34 (12.5)	4 (15.4)	0.674
Chronic lung disease	5 (1.8)	0 (0)	> 0.999
Chronic liver disease	13 (4.8)	1 (3.8)	> 0.999
Chronic kidney disease	2 (0.7)	0 (0)	> 0.999
Other organ cancer	25 (9.2)	2 (7.7)	0.799
Charlson comorbidity index, no (%)			
0	77 (28.3)	6 (23.1)	0.731
1	111 (40.8)	13 (50.0)	
≥ 2	84 (30.9)	7 (26.9)	
Family history of gastric cancer, no (%)	44 (16.2)	6 (23.1)	0.368
Use of antithrombotic drugs, no (%)			
No	199 (73.2)	16 (61.5)	0.328
Antiplatelet drugs	69 (25.4)	10 (38.5)	
Anticoagulant	4 (1.5)	0 (0)	
Tumor size (cm), mean ± SD	1.9 ± 1.2	1.9 ± 0.9	0.920
Tumor location, no (%)			
Upper	31 (11.4)	3 (11.5)	> 0.999
Middle	74 (27.2)	7 (26.9)	
Lower	167 (61.4)	16 (61.5)	
Tumor depth, no (%)			
Mucosa	222 (81.6)	25 (96.2)	0.060
Submucosa	50 (18.4)	1 (3.8)	
Tumor histologic type, no (%)			
Differentiated type	263 (96.7)	25 (96.2)	0.605
Undifferentiated type	9 (3.3)	1 (3.8)	
Initial multiple tumors, no (%)	28 (10.3)	4 (15.4)	0.423

Abbreviations: IQR, interquatile range; SD, standard deviation.

^a^ Cardiovascular diseases include coronary artery diseases (angina pectoris, myocardial infarction) and cerebrovascular diseases (cerebral hemorrhage and infarction).

### 3.4 Risk Factors for Metachronous Recurrence of Gastric Cancer

Univariate Cox‐proportional hazard regression analyses showed that only persistent *H. pylori* ‐positive infection status was a significant factor for MGC recurrence (hazard ratio [HR], 2.29; 95% confidence interval [CI]: 1.04–5.04) (Table 3). Age and sex‐adjusted HR for MGC recurrence in patients with persistently positive *H. pylori* infection was 2.33 (95% CI: 1.05–5.17).

**TABLE 3:** Risk factors associated with metachronous gastric cancer recurrence.

	Total no.	Univariate analysis			Multivariate analysis		
		cHR	95% CI	*p*	aHR	95% CI	*p*
Age group							
75–79	207	1.00			1.00		
≥ 80	91	0.93	0.39–2.23	0.878	0.84	0.35–2.03	0.704
Sex							
Female	97	1.00			1.00		
Male	201	1.07	0.47–2.46	0.871	1.06	0.46–2.44	0.897
*H. pylori* infection status							
Negative or eradicated	233	1.00			1.00		
Persistent	65	2.29	1.04–5.04	0.041	2.33	1.05–5.17	0.038
Charlson comorbidity index							
0	83	1.00					
1	124	1.24	0.47–3.25	0.669			
≥ 2	91	0.92	0.31–2.74	0.882			
Family history of gastric cancer	50	1.36	0.55–3.38	0.511			
Use of antithrombotic drugs							
No	215	1.00					
Yes	83	1.61	0.73–3.55	0.238			
Tumor size (cm)	298	1.03	0.74–1.42	0.876			
Tumor location							
Lower	183	1.00					
Middle	81	1.05	0.43–2.54	0.923			
Upper	34	1.05	0.31–3.62	0.936			
Tumor depth							
Mucosa	247	1.00					
Submucosa	51	0.22	0.03–1.64	0.140			
Tumor histologic type							
Differentiated type	288	1.00					
Undifferentiated type	10	1.30	0.18–9.60	0.798			
Initial multiple tumors	32	1.46	0.50–4.23	0.490			

Abbreviations: aHR, adjusted hazard ratio; cHR, crude hazard ratio; CI, confidence interval.

### 3.5 Overall survival After ESD

The 5‐year and 10‐year OS rates after ESD were 90.9% and 70.2%, respectively, with no gastric‐cancer‐related deaths. The OS in the *Hp*‐positive group did not differ compared with that in the *Hp*‐negative group (*p* = 0.102 by log‐rank test) (Figure S3).

## 4 Discussion

Patients with persistently positive *H. pylori* infection showed a higher incidence of MGC recurrence after ESD than those with negative *H. pylori* infection or eradicated status among late elderly EGC patients (aged ≥ 75 years). In late elderly EGC patients, a persistent *H. pylori* ‐positive infection is a risk factor associated with the MGC recurrence. In addition, a subgroup analysis involving patients with a baseline histological assessment of GA and IM showed that MGC recurrence was observed only in patients with advanced GA or IM stages (OLGA or OLGIM stage II to IV).

*H. pylori* infection is an important risk factor for MGC after ESD of EGC, and guidelines strongly recommend *H. pylori* eradication to prevent MGC after ESD [19, 20]. Globally, the *H. pylori* prevalence rate is 49% and tends to increase with age, with a prevalence of 56% in the older population (aged > 60 years) [21, 22]. In addition, older age is a risk factor for MGC after ESD of EGC [11]. Elderly patients with EGC had multiple risk factors for MGC, including age, high *H. pylori* infection rate, GA, and IM. Previous studies involving elderly patients with EGC were mainly focused on post‐ESD prognosis or procedure safety [7, 8, 9], but have not investigated the association between MGC and *H. pylori* infection. Our study involving late elderly EGC patients showed that persistently positive *H. pylori* infection was significantly associated with a higher MGC incidence after ESD. However, patients with successful *H. pylori* eradication did not show an increased incidence of MGC. Thus, *H. pylori* eradication might be effective in late elderly EGC patients aged ≥ 75 years to prevent MGC recurrence after ESD.

Previous studies (RCTs) reported that the gastric cancer prevention effect of *H. pylori* eradication was observed only in participants without precancerous lesions (GA, IM, or dysplasia) [23, 24]. Based on these results, the Japanese guideline and consensus report have recommended *H. pylori* eradication at a younger age [25, 26, 27]. Contrary to the results of the previous studies [23, 24], in other RCTs, the preventive effect of *H. pylori* eradication against gastric cancer was evident in older age groups (55–71 years) [28, 29]. The European *H. pylori* management guidelines also state that *H. pylori* eradication might provide a beneficial effect at any age in adulthood, and age is not a limiting factor for *H. pylori* treatment for gastric cancer prevention [20]. Our study showed a 2.3‐fold higher incidence of MGC after ESD in late elderly EGC patients with persistent *H. pylori* infection. Although our study did not evaluate the interaction effect between *H. pylori* infection and gastric premalignant lesions (GA and IM) due to limited information in approximately two‐thirds of patients, previous RCTs confirmed that *H. pylori* eradication reduced MGC recurrence in EGC patients with advanced GA and IM [13, 14]. Therefore, our study result provides evidence to justify the eradication of *H. pylori* for preventing MGC after ESD, even in late elderly EGC patients.

GA and IM are also important risk factors for MGCs [11]. The OLGA and OLGIM stages were useful systems to assess the extent of GA and IM [16, 17]. The two systems have shown good performance in discriminating risk groups for gastric neoplastic lesions (cancer and dysplasia) in previous case–control and cohort studies [30, 31, 32]. In previous cohort studies involving healthy participants or patients with EGC who underwent ESD, patients with OLGA or OLGIM stage II showed an increased risk of gastric neoplastic lesions [33, 34]. Like these results, subgroup analyses in our study also showed a higher MGC incidence in patients with OLGA or OLGIM stages II to IV, whereas no MGC recurrence was observed in those with stages 0 to I. Thus, in late elderly patients with EGC, endoscopic surveillance is needed during long‐term periods, particularly in those with advanced GA or IM even after *H. pylori* eradication.

Owing to their expected survival, physical status, and severe comorbidities (such as renal or liver dysfunction), elderly patients tend to experience AEs associated with *H. pylori* treatment [35]. Patient age, rather than the clinical situation, might affect clinicians' decisions on prescribing or withholding *H. pylori* treatment in elderly patients, unlike the situation in younger patients. However, *H. pylori* treatment regimens recommended by the guidelines showed similar efficacies and AEs in younger and late elderly patients (> 75 years) [35, 36]. Thus, if the patient's physical status is acceptable and the benefits of *H. pylori* treatment outweigh the potential harm, including AEs, clinicians might offer *H. pylori* treatment to late elderly patients regardless of age, particularly for conditions for which *H. pylori* eradication is strongly recommended, including peptic ulcer disease, gastric mucosa‐associated lymphoid tissue lymphoma, and post‐endoscopic resection status of EGC.

Our study evaluated the association between MGC recurrence and *H. pylori* infection status in many elderly EGC patients aged ≥ 75 years during a long‐term follow‐up. However, this study has several limitations. First, selection bias was inevitable due to the retrospective study design, and patients who did not meet inclusion criteria were excluded (no information on the follow‐up *H. pylori* status, short‐term follow‐up of < 1 year, history of gastrectomy, and noncurative resection). Second, not all patients were assessed for GA and IM. Although OLGA or OLGIM stages were associated with MGC recurrence in the subgroup analyses, these results need to be confirmed by further studies. Third, the *H. pylori* treatment effect on MGC recurrence was not directly compared such as that in interventional RCTs, although *H. pylori* ‐eradicated patients had a lower incidence of metachronous recurrence than those with a persistent *H. pylori* ‐positive status. However, it might be unethical to perform an RCT involving late elderly patients with EGC because the guidelines strongly recommend *H. pylori* treatment after ESD. Fourth, the initiation time point for the follow‐up to assess study outcomes was the date of ESD. Therefore, in the *H. pylori* ‐eradicated patients, there might be about a 2‐ to 6‐month gap between the ESD date and the confirmation date of *H. pylori* eradication success. Finally, the payment policy for the cost of *H. pylori* treatment (100% cost by patients before 2018 and almost covered by the Korean National Health Insurance Service after 2018) differed during the study periods. This change might affect the decision of *H. pylori* treatment.

In conclusion, persistent *H. pylori* ‐positive infection status was associated with MGC recurrence in EGC patients aged ≥ 75 years who underwent ESD. *H. pylori* treatment should be provided to late elderly EGC patients after ESD without consideration of chronological age if the patient's condition is conducive.

## Supporting information


**Figure S1:** Incidence of metachronous gastric cancer in the *H. pylori* ‐eradicated patients and persistent patients.
**Figure S2:** Incidence of metachronous gastric cancer in the *H. pylori* ‐negative patients and eradicated patients.
**Figure S3:** Overall survival after ESD according to the *H. pylori* infection status.
**Table S1:** Comparison of baseline patient and tumor characteristics according to study inclusion.
**Table S2:** Tumor characteristics and treatment of metachronous gastric cancer.

## Data Availability

The data that support the findings of this study are available on request from the corresponding author. The data are not publicly available due to privacy or ethical restrictions.

## References

[1] United Nations, Department of Economic and Social Affairs, Population Division , “World Population Prospects 2024: Summary of Results,” (2024), https://population.un.org/wpp/assets/Files/WPP2024_Summary‐of‐Results.pdf.

[2] United Nations, Department of Economic and Social Affairs, Population Division , “World Population Ageing 2019: Highlights (ST/ESA/SER.A/430),” (2019), https://digitallibrary.un.org/record/3846855?v=pdf.

[3] J. Ferlay , M. Ervik , F. Lam , et al., Global Cancer Observatory: Cancer Today (International Agency for Research on Cancer, 2024), https://gco.iarc.who.int/today.

[4] I. H. Kim , S. J. Kang , W. Choi , et al., “Korean Practice Guidelines for Gastric Cancer 2024: An Evidence‐Based, Multidisciplinary Approach (Update of 2022 Guideline),” Journal of Gastric Cancer 25 (2025): 5–114. 39822170 10.5230/jgc.2025.25.e11 PMC11739648

[5] Japanese Gastric Cancer Association , “Japanese Gastric Cancer Treatment Guidelines 2021 (6th Edition),” Gastric Cancer 26 (2023): 1–25. 36342574 10.1007/s10120-022-01331-8 PMC9813208

[6] R. Ofori‐Asenso , K. L. Chin , A. J. Curtis , E. Zomer , S. Zoungas , and D. Liew , “Recent Patterns of Multimorbidity Among Older Adults in High‐Income Countries,” Population Health Management 22 (2019): 127–137. 30096023 10.1089/pop.2018.0069

[7] M. Sekiguchi , I. Oda , H. Suzuki , et al., “Clinical Outcomes and Prognostic Factors in Gastric Cancer Patients Aged ≥85 Years Undergoing Endoscopic Submucosal Dissection,” Gastrointestinal Endoscopy 85 (2017): 963–972. 27751873 10.1016/j.gie.2016.10.013

[8] K. Waki , S. Shichijo , N. Uedo , et al., “Long‐Term Outcomes After Endoscopic Resection for Late‐Elderly Patients With Early Gastric Cancer,” Gastrointestinal Endoscopy 95 (2022): 873–883. 34979116 10.1016/j.gie.2021.12.028

[9] C. H. Park , H. Lee , D. W. Kim , et al., “Clinical Safety of Endoscopic Submucosal Dissection Compared With Surgery in Elderly Patients With Early Gastric Cancer: A Propensity‐Matched Analysis,” Gastrointestinal Endoscopy 80 (2014): 599–609. 24973177 10.1016/j.gie.2014.04.042

[10] K. Miyahara , M. Ishida , Y. Kono , et al., “Prognosis After Curative Resection for Stage IA Gastric Cancer in Elderly Patients: Endoscopic Submucosal Dissection Versus Surgery,” Surgery Today 52 (2022): 1329–1340. 35089444 10.1007/s00595-022-02456-0

[11] R. Ortigão , G. Figueirôa , L. Frazzoni , et al., “Risk Factors for Gastric Metachronous Lesions After Endoscopic or Surgical Resection: A Systematic Review and Meta‐Analysis,” Endoscopy 54 (2022): 892–901. 35104897 10.1055/a-1724-7378

[12] A. C. Ford , Y. Yuan , and P. Moayyedi , “ *Helicobacter pylori* Eradication Therapy to Prevent Gastric Cancer: Systematic Review and Meta‐Analysis,” Gut 69 (2020): 2113–2121. 32205420 10.1136/gutjnl-2020-320839

[13] K. Fukase , M. Kato , S. Kikuchi , et al., “Effect of Eradication of *Helicobacter pylori* on Incidence of Metachronous Gastric Carcinoma After Endoscopic Resection of Early Gastric Cancer: An Open‐Label, Randomised Controlled Trial,” Lancet 372 (2008): 392–397. 18675689 10.1016/S0140-6736(08)61159-9

[14] I. J. Choi , M. C. Kook , Y. I. Kim , et al., “ *Helicobacter pylori* Therapy for the Prevention of Metachronous Gastric Cancer,” New England Journal of Medicine 378 (2018): 1085–1095. 29562147 10.1056/NEJMoa1708423

[15] R. A. Deyo , D. C. Cherkin , and M. A. Ciol , “Adapting a Clinical Comorbidity Index for Use With ICD‐9‐CM Administrative Databases,” Journal of Clinical Epidemiology 45 (1992): 613–619. 1607900 10.1016/0895-4356(92)90133-8

[16] M. Rugge , P. Correa , F. Di Mario , et al., “OLGA Staging for Gastritis: A Tutorial,” Digestive and Liver Disease 40 (2008): 650–658. 18424244 10.1016/j.dld.2008.02.030

[17] L. G. Capelle , A. C. de Vries , J. Haringsma , et al., “The Staging of Gastritis With the OLGA System by Using Intestinal Metaplasia as an Accurate Alternative for Atrophic Gastritis,” Gastrointestinal Endoscopy 71 (2010): 1150–1158. 20381801 10.1016/j.gie.2009.12.029

[18] S. J. Cho , I. J. Choi , C. G. Kim , et al., “Aspirin Use and Bleeding Risk After Endoscopic Submucosal Dissection in Patients With Gastric Neoplasms,” Endoscopy 44 (2012): 114–121. 22271021 10.1055/s-0031-1291459

[19] H. K. Jung , S. J. Kang , Y. C. Lee , et al., “Evidence‐Based Guidelines for the Treatment of *Helicobacter pylori* Infection in Korea 2020,” Gut Liver 15 (2021): 168–195. 33468712 10.5009/gnl20288 PMC7960974

[20] P. Malfertheiner , F. Megraud , T. Rokkas , et al., “Management of *Helicobacter pylori* Infection: The Maastricht VI/Florence Consensus Report,” Gut 71 (2022): 1724–1762. 10.1136/gutjnl-2022-327745 35944925

[21] Y. C. Chen , P. Malfertheiner , H. T. Yu , et al., “Global Prevalence of *Helicobacter pylori* Infection and Incidence of Gastric Cancer Between 1980 and 2022,” Gastroenterology 166 (2024): 605–619. 38176660 10.1053/j.gastro.2023.12.022

[22] Y. Li , H. Choi , K. Leung , F. Jiang , D. Y. Graham , and W. K. Leung , “Global Prevalence of *Helicobacter pylori* Infection Between 1980 and 2022: A Systematic Review and Meta‐Analysis,” Lancet Gastroenterology & Hepatology 8 (2023): 553–564. 37086739 10.1016/S2468-1253(23)00070-5

[23] B. C. Wong , S. K. Lam , W. M. Wong , et al., “ *Helicobacter pylori* Eradication to Prevent Gastric Cancer in a High‐Risk Region of China: A Randomized Controlled Trial,” JAMA 291 (2004): 187–194. 14722144 10.1001/jama.291.2.187

[24] L. Yan , Y. Chen , F. Chen , et al., “Effect of *Helicobacter pylori* Eradication on Gastric Cancer Prevention: Updated Report From a Randomized Controlled Trial With 26.5 Years of Follow‐Up,” Gastroenterology 163 (2022): 154–162. 35364066 10.1053/j.gastro.2022.03.039

[25] J. M. Liou , P. Malfertheiner , Y. C. Lee , et al., “Screening and Eradication of *Helicobacter pylori* for Gastric Cancer Prevention: The Taipei Global Consensus,” Gut 69 (2020): 2093–2112. 33004546 10.1136/gutjnl-2020-322368

[26] K. Sugano , J. Tack , E. J. Kuipers , et al., “Kyoto Global Consensus Report on *Helicobacter pylori* Gastritis,” Gut 64 (2015): 1353–1367. 26187502 10.1136/gutjnl-2015-309252 PMC4552923

[27] M. Kato , H. Ota , M. Okuda , et al., “Guidelines for the Management of *Helicobacter pylori* Infection in Japan: 2016 Revised Edition,” Helicobacter 24 (2019): e12597. 31111585 10.1111/hel.12597

[28] W. Q. Li , J. L. Ma , L. Zhang , et al., “Effects of *Helicobacter pylori* Treatment on Gastric Cancer Incidence and Mortality in Subgroups,” Journal of the National Cancer Institute 106 (2014): dju116. 24925350 10.1093/jnci/dju116 PMC4067110

[29] W. Q. Li , J. Y. Zhang , J. L. Ma , et al., “Effects of *Helicobacter pylori* Treatment and Vitamin and Garlic Supplementation on Gastric Cancer Incidence and Mortality: Follow‐Up of a Randomized Intervention Trial,” BMJ 366 (2019): l5016. 31511230 10.1136/bmj.l5016 PMC6737461

[30] H. Yue , L. Shan , and L. Bin , “The Significance of OLGA and OLGIM Staging Systems in the Risk Assessment of Gastric Cancer: A Systematic Review and Meta‐Analysis,” Gastric Cancer 21 (2018): 579–587. 29460004 10.1007/s10120-018-0812-3

[31] M. Rugge , A. Meggio , C. Pravadelli , et al., “Gastritis Staging in the Endoscopic Follow‐Up for the Secondary Prevention of Gastric Cancer: A 5‐Year Prospective Study of 1755 Patients,” Gut 68 (2019): 11–17. 29306868 10.1136/gutjnl-2017-314600

[32] W. J. den Hollander , I. L. Holster , C. M. den Hoed , et al., “Surveillance of Premalignant Gastric Lesions: A Multicentre Prospective Cohort Study From Low Incidence Regions,” Gut 68 (2019): 585–593. 29875257 10.1136/gutjnl-2017-314498

[33] J. W. J. Lee , F. Zhu , S. Srivastava , et al., “Severity of Gastric Intestinal Metaplasia Predicts the Risk of Gastric Cancer: A Prospective Multicentre Cohort Study (GCEP),” Gut 71 (2022): 854–863. 33975867 10.1136/gutjnl-2021-324057 PMC8995828

[34] Y. S. Na , S. G. Kim , and S. J. Cho , “Risk Assessment of Metachronous Gastric Cancer Development Using OLGA and OLGIM Systems After Endoscopic Submucosal Dissection for Early Gastric Cancer: A Long‐Term Follow‐Up Study,” Gastric Cancer 26 (2023): 298–306. 36609936 10.1007/s10120-022-01361-2

[35] H. Gong , H. M. Xu , and D. K. Zhang , “Focusing on *Helicobacter pylori* Infection in the Elderly,” Frontiers in Cellular and Infection Microbiology 13 (2023): 1121947. 36968116 10.3389/fcimb.2023.1121947 PMC10036784

[36] S. Kobayashi , S. Joshita , C. Yamamoto , et al., “Efficacy and Safety of Eradication Therapy for Elderly Patients With *helicobacter pylori* Infection,” Medicine 98 (2019): e16619. 31348311 10.1097/MD.0000000000016619 PMC6709141

