# Supplementary material for: Helicobacter pylori Infection and Metachronous Gastric Cancer in Elderly Patients With Gastric Cancer Aged ≥ 75 Years Who Underwent Endoscopic Submucosal Dissection
Source: Helicobacter. 2025 Sep 1;30(4):e70068. doi: 10.1111/hel.70068 (PMC12402296; doi:10.1111/hel.70068)
Supplement: Supplementary file 1 — Figure S1: Incidence of metachronous gastric cancer in the H. pylori ‐eradicated patients and persistent patients. Figure S2: Incidence of metachronous gastric cancer in the H. pylori ‐negative patients and eradicated patients. Figure S3: Overall survival after ESD according to the H. pylori infection status. Table S1: Comparison of baseline patient and tumor characteristics according to study inclusion. Table S2: Tumor characteristics and treatment of metachronous gastric cancer. [file HEL-30-e70068-s001.docx]

**Supplementary materials**

**Long-term outcomes of endoscopic submucosal dissection vs. surgery in elderly early gastric cancer patients aged ≥75 years meeting the curative resection criteria**

**Table of Contents**

**Supplementary Figures**

Figure S1. Incidence of metachronous gastric cancer in the *H. pylori*-eradicated patients and -persistent patients ………………………………………………………………………………………………………3

Figure S2. Incidence of metachronous gastric cancer in the *H. pylori*-negative patients and -eradicated patients ………………………………………………………………………………………………………4

Figure S3. Overall survival after ESD according to the *H. pylori* infection status………………………………5

**Supplementary Tables**

Table S1. Comparison of baseline patient and tumor characteristics according to study inclusion…………….6

Table S2. Tumor characteristics and treatment of metachronous gastric cancer……………….……………….7

Figure S1. Incidence of metachronous gastric cancer in the *H. pylori*-eradicated patients and -persistent patients


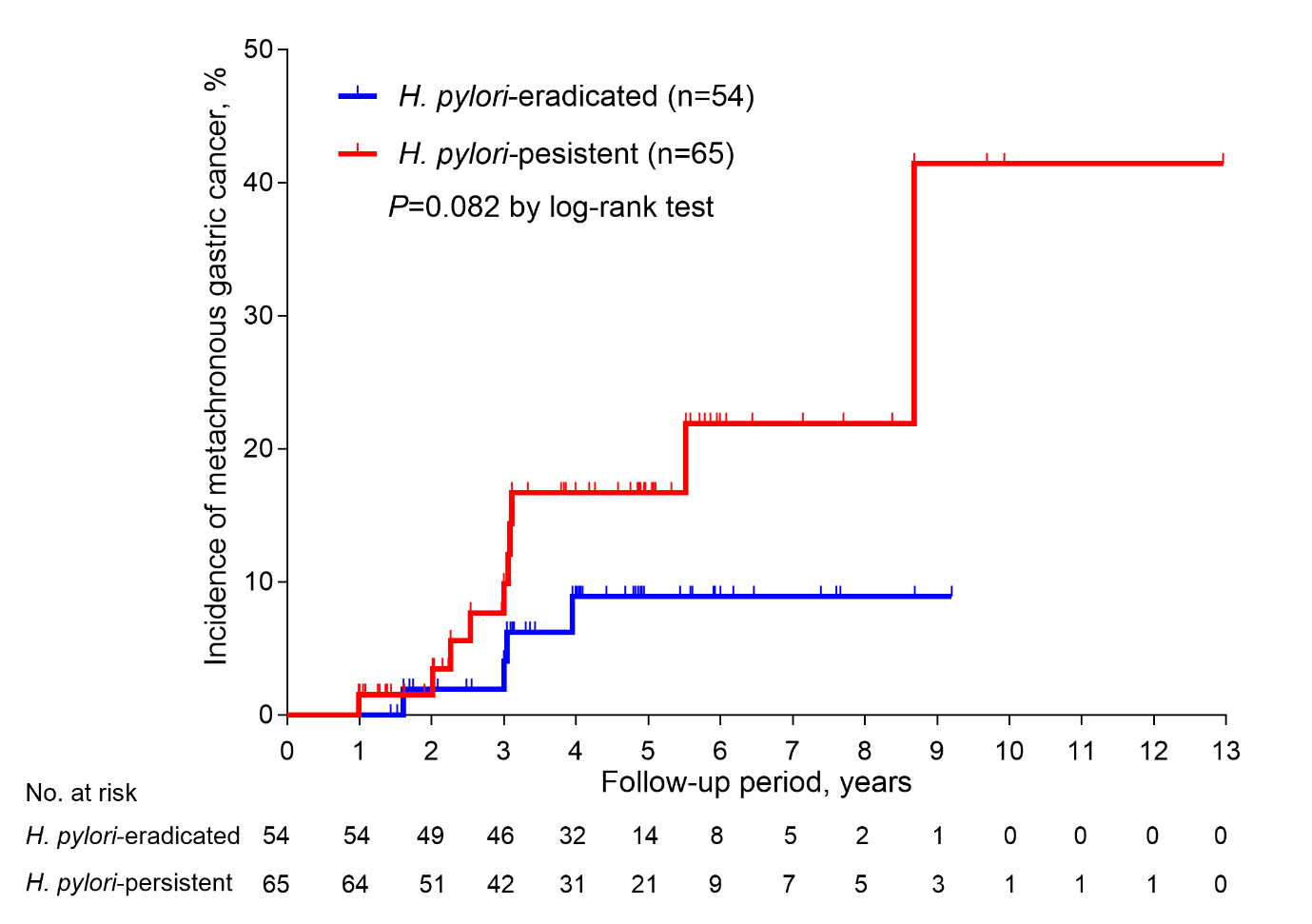


Figure S2. Incidence of metachronous gastric cancer in the *H. pylori*-negative patients and -eradicated patients


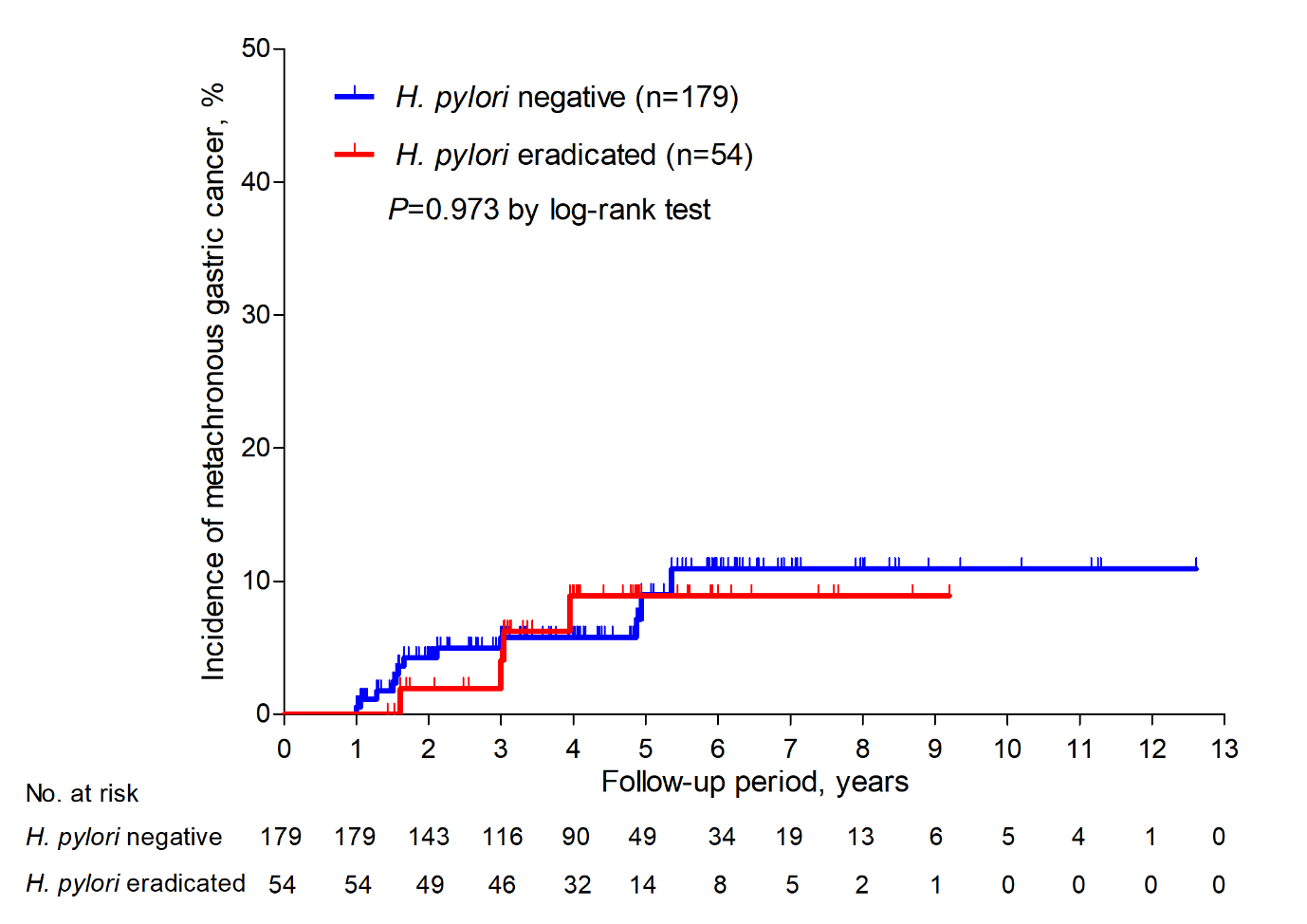


Figure S3. Overall survival after ESD according to the *H. pylori* infection status


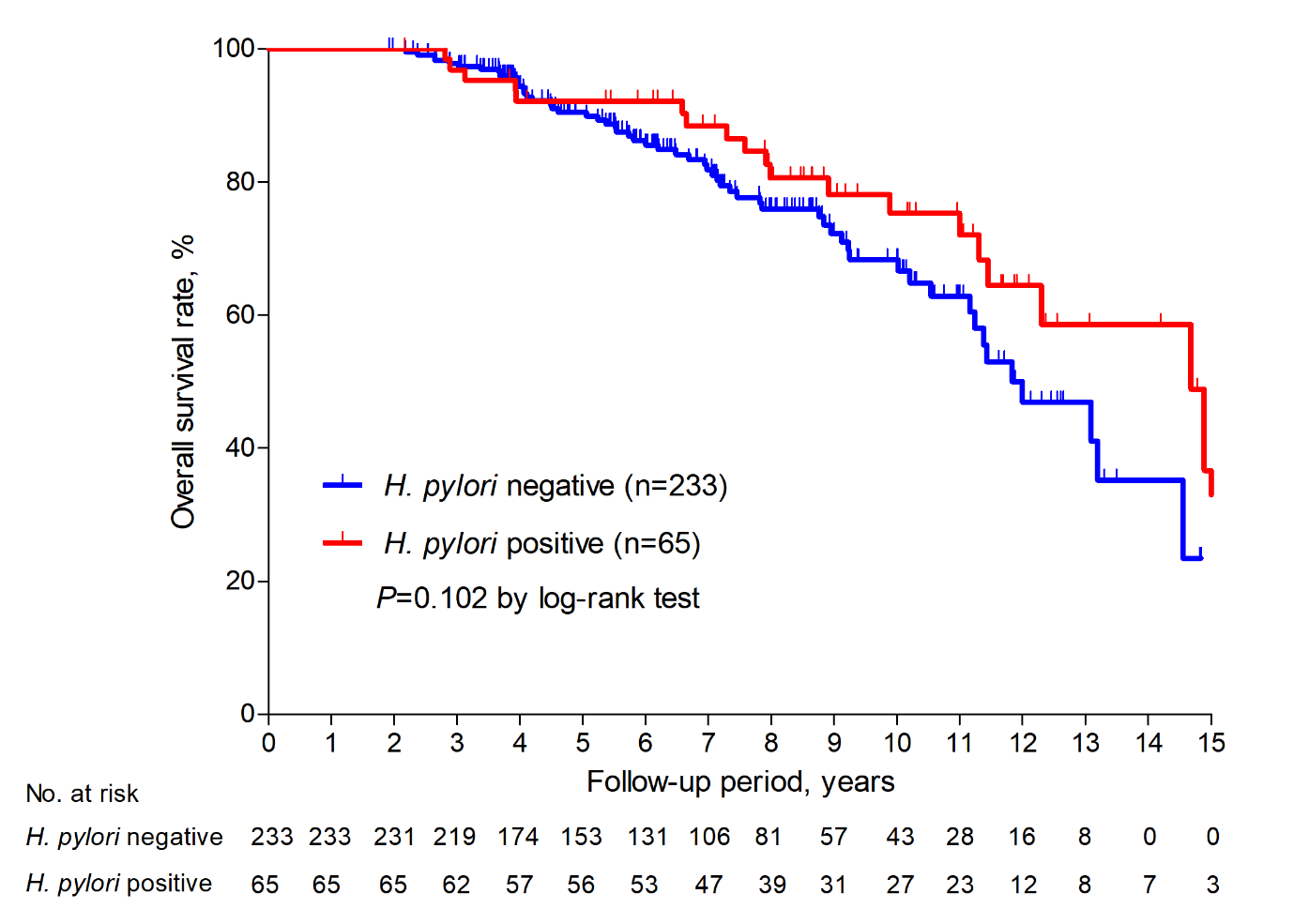


Table S1. Comparison of baseline patient and tumor characteristics according to study inclusion

|  | Included patients | Excluded patients | *P* |  |
| --- | --- | --- | --- | --- |
|  |  |  |  |  |
|  | (n=298) | (n=280) |  |  |
| Age (year), median (IQR) | 78 (76-80) | 78 (76-81) | 0.377 |  |
| Sex, no (%) |  |  | 0.990 |  |
| Female | 97 (32.6) | 91 (32.5) |  |  |
| Male | 201 (67.4) | 189 (67.5) |  |  |
| Co-morbid illness, no (%) |  |  |  |  |
| Hypertension | 158 (53.0) | 169 (60.4) | 0.075 |  |
| Diabetes mellitus | 65 (21.8) | 70 (25.0) | 0.365 |  |
| Atrial fibrillation | 12 (4.0) | 5 (1.8) | 0.111 |  |
| Cardiovascular disease* | 38 (12.8) | 40 (14.3) | 0.590 |  |
| Chronic lung disease | 5 (1.7) | 3 (1.1) | 0.533 |  |
| Chronic liver disease | 14 (4.7) | 10 (3.6) | 0.497 |  |
| Chronic kidney disease | 2 (0.7) | 4 (1.4) | 0.369 |  |
| Other organ cancer | 27 (9.1) | 4 (1.4) | <0.001 |  |
| Two or more co-morbid illnesses | 81 (27.2) | 101 (36.1) | 0.021 |  |
| CCI score, no (%) |  |  | <0.001 |  |
| 0 | 83 (27.9) | 149 (53.2) |  |  |
| 1 | 124 (41.6) | 105 (37.5) |  |  |
| ≥ 2 | 91 (30.5) | 26 (9.3) |  |  |
| Family history of gastric cancer, no (%) | 50 (16.8) | 60 (21.4) | 0.155 |  |
| Tumor size (cm), mean±SD | 1.9±1.2 | 2.1±1.4 | 0.079 |  |
| Tumor location, no (%) |  |  | 0.054 |  |
| Upper | 34 (11.4) | 33 (11.8) |  |  |
| Middle | 81 (27.2) | 57 (20.4) |  |  |
| Lower | 183 (61.4) | 186 (66.4) |  |  |
| Remnant stomach | 0 (0) | 4 (1.4) |  |  |
| Tumor depth, no (%) |  |  | <0.001 |  |
| Mucosa | 247 (82.9) | 183 (65.4) |  |  |
| Submucosa ≤ 500 um | 51 (17.1) | 95 (33.9) |  |  |
| Submucosa > 500 um or deeper | 0 (0) | 2 (0.7) |  |  |
| Tumor histologic type, no (%) |  |  | 0.027 |  |
| Differentiated type | 288 (96.6) | 259 (92.5) |  |  |
| Undifferentiated type | 10 (3.4) | 21 (7.5) |  |  |
| Initial multiple tumors, no (%) | 32 (10.7) | 17 (6.1) | 0.044 |  |
| Abbreviation: SD, standard deviation; IQR, interquartile range ; CCI, Charlson comorbidity index. | | | | |
| *Cardiovascular diseases include coronary artery diseases (angina pectoris, myocardial infarction) and cerebrovascular diseases (cerebral hemorrhage and infarction). | | | | |

Table S2. Tumor characteristics and treatment of metachronous gastric cancer

|  | Total no. = 26 |
| --- | --- |
| Age at diagnosis (year), median (IQR) | 80 (78-84) |
| Sex, no (%) |  |
| Female | 8 (30.8) |
| Male | 18 (69.2) |
| Time to metachronous recurrence (year), median (IQR) | 2.8 (1.6-3.1) |
| *H. pylori* infection status, no (%) |  |
| Negative | 12 (46.2) |
| Eradicated | 4 (15.4) |
| Positive | 10 (38.5) |
| Tumor size (cm), mean±SD | 1.35±0.78 |
| Tumor location, no (%) |  |
| Upper | 3 (11.5) |
| Middle | 7 (26.9) |
| Lower | 16 (61.5) |
| Tumor depth, no (%) |  |
| Mucosa | 25 (96.2) |
| Submucosa | 1 (3.8) |
| Tumor histologic type, no (%) |  |
| Well differentiated tubular adenocarcinoma | 25 (96.2) |
| Moderately differentiated tubular adenocarcinoma | 1 (3.8) |
| Treatment, no (%) |  |
| Argon plasma coagulation | 1 (3.8) |
| ESD | 23 (88.5) |
| Surgery | 2 (7.7) |
| Abbreviations: IQR, interquatile range; SD, standard deviation; ESD, endoscopic submucosal dissection. | |
